# High-fidelity entanglement swapping and generation of three-qubit GHZ state using asynchronous telecom photon pair sources

**DOI:** 10.1038/s41598-018-19738-8

**Published:** 2018-01-23

**Authors:** Yoshiaki Tsujimoto, Motoki Tanaka, Nobuo Iwasaki, Rikizo Ikuta, Shigehito Miki, Taro Yamashita, Hirotaka Terai, Takashi Yamamoto, Masato Koashi, Nobuyuki Imoto

**Affiliations:** 1 Graduate School of Engineering Science, Osaka University, Toyonaka, Osaka 560-8531 Japan; 2 Advanced ICT Research Institute, National Institute of Information and Communications Technology (NICT), Koganei, Tokyo 184-8795 Japan; 3 Advanced ICT Research Institute, National Institute of Information and Communications Technology (NICT), Kobe, 651-2492 Japan; 4 Photon Science Center, The University of Tokyo, Bunkyo-ku, 113-8656 Japan

## Abstract

We experimentally demonstrate a high-fidelity entanglement swapping and a generation of the Greenberger-Horne-Zeilinger (GHZ) state using polarization-entangled photon pairs at telecommunication wavelength produced by spontaneous parametric down conversion with continuous-wave pump light. While spatially separated sources asynchronously emit photon pairs, the time-resolved photon detection guarantees the temporal indistinguishability of photons without active timing synchronizations of pump lasers and/or adjustment of optical paths. In the experiment, photons are sufficiently narrowed by fiber-based Bragg gratings with the central wavelengths of 1541 nm & 1580 nm, and detected by superconducting nanowire single-photon detectors with low timing jitters. The observed fidelities of the final states for entanglement swapping and the generated three-qubit state were 0.84 ± 0.04 and 0.70 ± 0.05, respectively.

## Introduction

Entanglement swapping^1^ is an entangling operation on two independent photons and a key technique for implementing various quantum information processing such as quantum repeaters^2^, and quantum computation^3^. In order to perform such tasks successfully, indistinguishability of the independently generated photons is of importance. Spontaneous parametric down conversion (SPDC) is a standard method to generate entangled photon pairs and many experiments of entanglement swapping utilizing SPDC have been demonstrated^4–9^. In such experiments, SPDC photons are generated by using ultrafast pulsed lasers, and temporal indistinguishability of the photons is provided through a timing synchronization of the photon generation and/or the precise stabilization of the optical path lengths. The alternative way to provide temporal indistinguishability is employing continuous wave (cw) pumped photon pair sources and the photodetectors whose temporal resolution is much shorter than the coherence time of the photons, which is realized by increasing coherence time of photons with narrow bandpass filters and/or employing photodetectors with low timing jitters^10–14^. This method mitigates accuracy of timing synchronization of photons, and will be useful for a long-distance quantum communication. In addition, the narrow bandwidth of the photons allows dense frequency multiplexing.

The entanglement swapping using asynchronous photon pair source is firstly demonstrated by Halder *et al*.^10^, in which they utilized energy-time entangled photon pairs at telecom wavelengths generated by cw-pumped SPDC and photodetectors with low timing jitters. In the demonstration, an observed visibility and a four-fold count rate are 0.63 ± 0.02 and 5 counts/hour, respectively, which are much smaller than those observed with a timing synchronization of pulse-pumped SPDC^5–9^. Higher efficiency and visibility will be desirable for performing various kinds of applications such as quantum repeaters^2^, quantum relays^15,16^, measurement-device-independent quantum key distribution^17–19^ and distributed quantum computation^20^.

In this paper, we show high-fidelity and extended entanglement manipulation using telecom-band asynchronous polarization entangled photon pairs. First we demonstrate high-fidelity entanglement swapping using polarization entangled photon pairs generated by cw-pumped SPDC with high-resolution photon detectors. We performed quantum state tomography (QST) on swapped photon pairs and reconstructed its density operator. The observed fidelity is 0.84 ± 0.04, which is much higher than the previously reported value, and as high as those observed by using pulse-pumped SPDC. Second, we for the first time demonstrated generation of a telecom-band three-qubit Greenberger-Horne-Zeilinger (GHZ) state using asynchronous sources, and observed a fidelity of 0.70 ± 0.05, which is applicable to quantum communication using multipartite entanglement such as secret sharing^21^ and quantum cryptography^22^. In our experiments, the efficiencies are also highly improved compared to the previous experiment^10^. We obtain the four-fold coincidence rates of 28 counts/hour and 131 counts/hour for entanglement swapping and GHZ state generation, respectively. Furthermore, by enlarging the width of each coincidence window to be as large as the coherence time of SPDC photons, we still kept a high fidelity of 0.75 ± 0.02 for entanglement swapping with a four-fold coincidence rate of as high as 400 counts/hour. These results pave the way for high-quality and efficient photonic quantum information processing in cw regime.

## Experiment and Results

### Experimental setup

**Figure 1:**
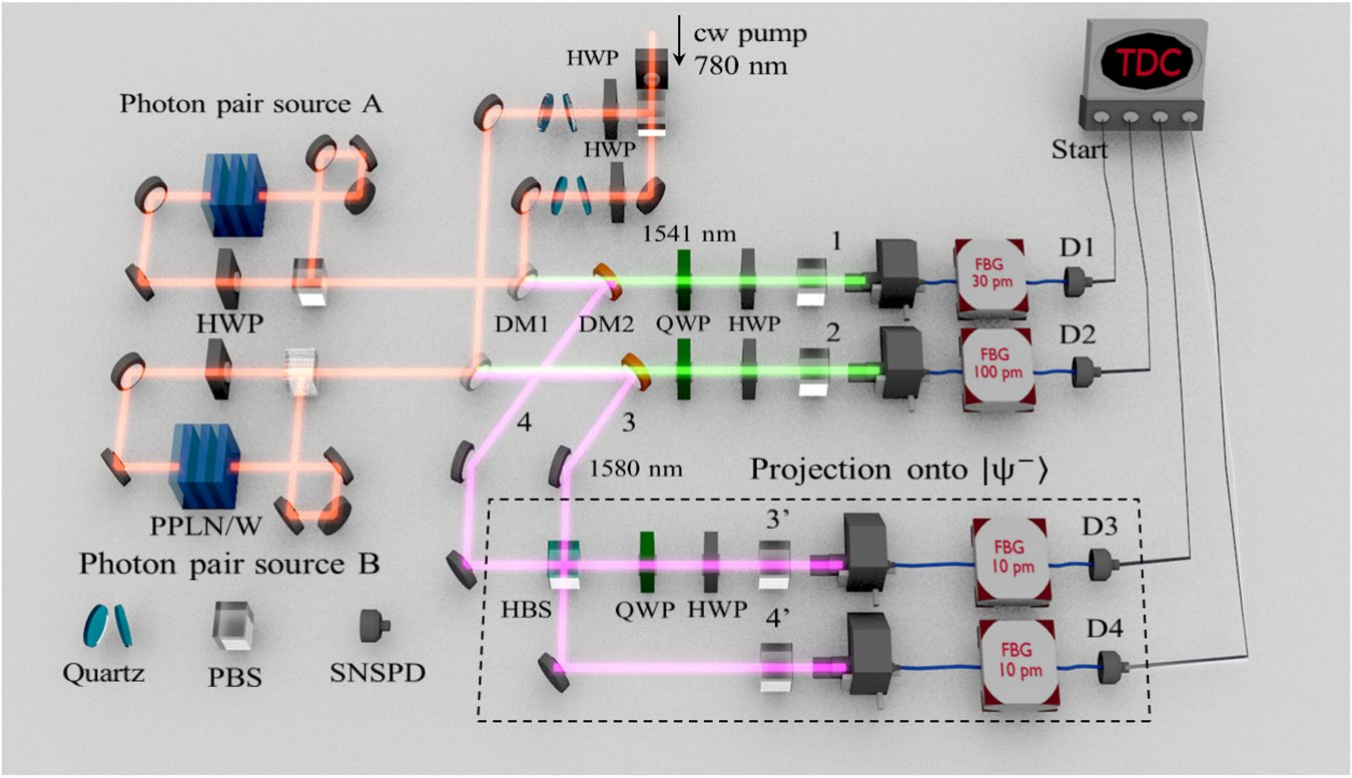
Experimental setup. The cw pump beam at 780 nm is obtained by the second-harmonic generation based on a periodically-poled lithium niobate waveguide (PPLN/W). An entangled photon pair at 1541 nm and 1580 nm is generated by PPLN/W in a Sagnac configuration. After projecting the polarization state of photons at 1580 nm, the photons at 1541 nm become entangled.

The experimental setup is shown in Fig. 1. A cw pump beam at 780 nm is obtained by second-harmonic generation of light at 1560 nm from an external cavity diode laser with a linewidth of 1.8 kHz^14^. At photon pair source A, an entangled photon pair $|{\varphi }^{+}\rangle ={(|{\rm{H}}\rangle }_{1541}|{\rm{H}}{\rangle }_{1580}+|{\rm{V}}{\rangle }_{1541}|{\rm{V}}{\rangle }_{1580})/\sqrt{2}$ at 1541 nm and 1580 nm is generated by a 40-mm-long and type-0 quasi-phase-matched periodically-poled lithium niobate waveguide (PPLN/W) in a Sagnac configuration with a polarizing beamsplitter (PBS), where |H〉 and |V〉 represent horizontal (H) and vertical (V) polarization states of a photon, respectively. The pump beam is removed from the generated SPDC photons by dichroic mirror 1 (DM1). DM2 divides spatial modes of the two photons at 1541 nm and 1580 nm into modes 1 and 4, respectively. Similarly, we prepared the other entangled photon pair at 1541 nm in mode 2 and 1580 nm in mode 3 at photon pair source B in Fig. 1. Photons at 1580 nm in mode 3 and mode 4 are mixed by a half beamsplitter (HBS). The coincidence detection between mode 3′ with V-polarization and mode 4′ with H-polarization is regarded as a projection of a photon pair in modes 3 and 4 into the singlet state $|{\psi }^{-}\rangle =(|{\rm{HV}}\rangle -|{\rm{VH}}\rangle )/\sqrt{2}$ ideally. As a result, the polarization state in modes 1 and 2 also becomes |*ψ*^−^〉. In order to improve the indistinguishability of the photons in modes 3′ and 4′, the photons are filtered by fiber-based Bragg gratings (FBGs) with bandwidths of 10 pm followed by superconducting nanowire single-photon detectors (SNSPDs) whose timing jitter is *τ*_*j*_ = 85 ps each^14^. The photons in modes 1 and 2 are also filtered by FBGs with bandwidths of 30 pm and 100 pm, respectively, which plays a role of avoiding saturation of the single counts of SNSPDs. Assuming that the transmission spectra of FBGs are Gaussians with FWHMs of 10 pm, 30 pm and 100 pm, the corresponding temporal widths of single-photon wave packets are calculated to be *τ*_3′_ = *τ*_4′_ = 367 ps, *τ*_1_ = 116 ps and *τ*_2_ = 35 ps, respectively. We estimate the coherence time *τ*_*c*_ of the SPDC photon pair to be ${\tau }_{c}=\sqrt{{\tau }_{\mathrm{1(2)}}^{2}+{\tau }_{3^{\prime} }^{2}}=385$ (369) ps for the photon pair source A (B). In the experiment, *τ*_*c*_ is estimated by the temporal distribution of two-fold coincidence events of the photon pairs. Assuming that the temporal distribution is determined by three Gasussians with FWHMs of *τ*_*j*_, *τ*_*j*_, and *τ*_*c*_, we estimated *τ*_*c*_ to be about 231 ps (183 ps) FWHM for the photon pair source A (B). The experimental values of *τ*_*c*_ are less than those expected from the bandwidths of the FBSs. We guess the reason is the actual transmission spectra of the FBSs are non-Gaussian. Nonetheless the condition ${\tau }_{c}\gg {\tau }_{j}$ for a high-visibility interference^10,14^ is still satisfied. The electric signal from D1 is connected to a time-to-digital converter (TDC) as a start signal, and the electric signals from D2, D3 and D4 are used as the stop signals of the TDC. We collect all of timestamps of the stop signals for every start signal with time slot of 1 ps. We postselect the records of the three stop signals within time windows *τ*_w_. If we find at least one detection event in each of the three stop signals, we regard it as a four-fold coincidence event.

### Entanglement swapping

**Figure 2:**
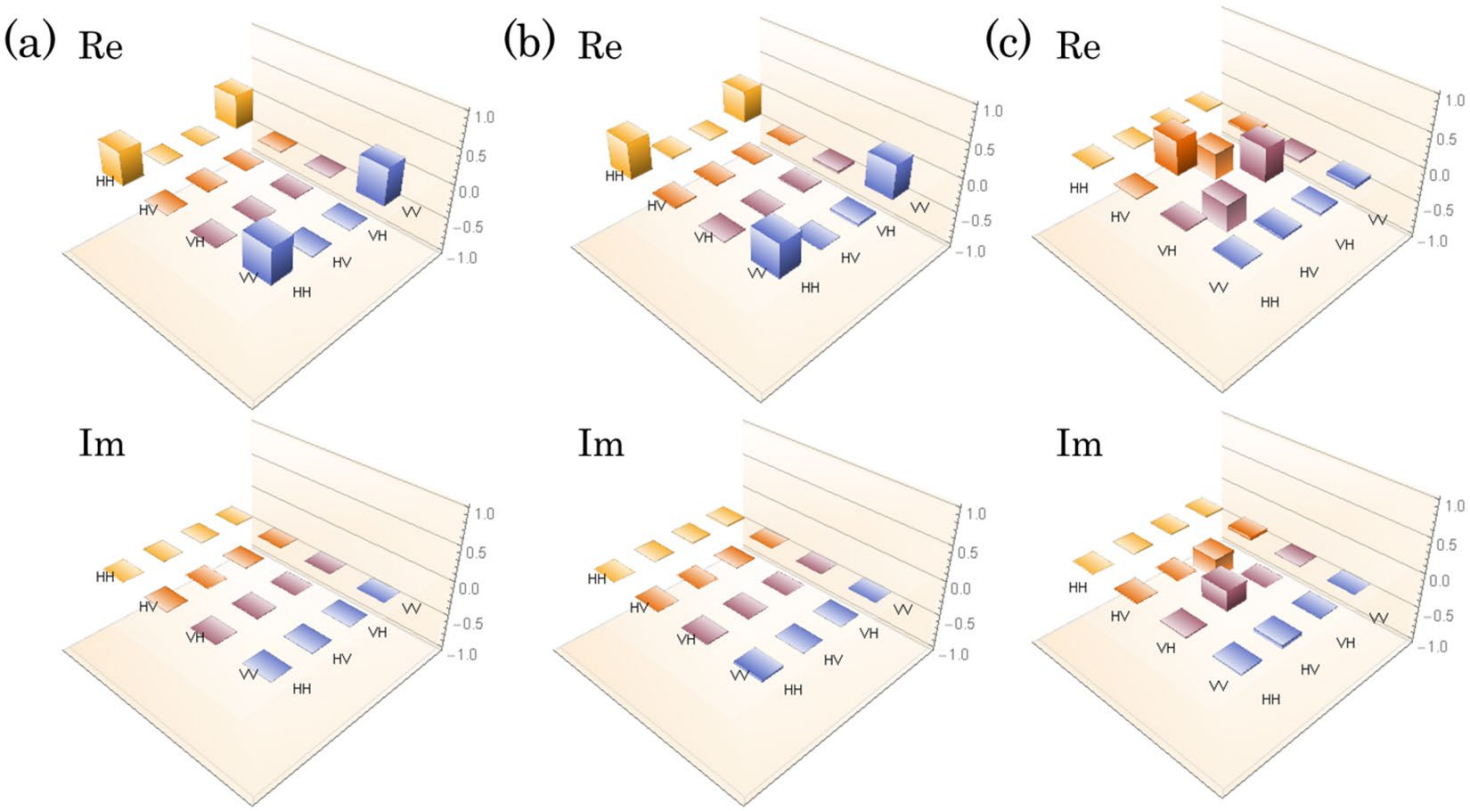
The real parts and imaginary parts of the density operators of (**a**) ${\hat{\rho }}_{A}$, (**b**) ${\hat{\rho }}_{B}$ and (**c**) ${\hat{\rho }}_{{\rm{swap}}}$.

Before performing entanglement swapping, we first characterized the initial entangled photon pairs from photon pair source A (${\hat{\rho }}_{A}$) and photon pair source B (${\hat{\rho }}_{B}$) by measuring the two-fold coincidence count between D1 & D3, and D2 & D3, respectively. By performing the QST and diluted maximum-likelihood algorithm^23^, we reconstructed the density operators as shown in Fig. 2(a,b). Observed fidelities defined by $\langle {\varphi }^{+}|{\hat{\rho }}_{A}|{\varphi }^{+}\rangle$ and $\langle {\varphi }^{+}|{\hat{\rho }}_{B}|{\varphi }^{+}\rangle$ were 0.963 ± 0.004 and 0.931 ± 0.004, respectively, which implies that the two photon pairs are highly entangled. The detection rates of ${\hat{\rho }}_{A}$ and ${\hat{\rho }}_{B}$ calculated by the sum of the coincidence rates for HH, HV, VH and VV basis measurement were 5.1 kHz and 5.2 kHz, respectively, with *τ*_w_ = 80 ps and 3.5 mW pump power for clockwise and counterclockwise directions of the Sagnac interferometers.

Next, we performed the entanglement swapping. We postselect the detection events of D1 and D2 such that the heralded single photons in modes 3 and 4 become temporally indistinguishable. We reconstructed the density operator ${\hat{\rho }}_{{\rm{swap}}}$ of the photon pairs in modes 1 and 2 by using the detection events in which the four-fold coincidence among D1, D2, D3 and D4 occurs. The four-fold coincidence rate calculated by the sum of the four-fold coincidence rates for HH, HV, VH and VV basis measurement was 28 counts/hour with *τ*_w_ = 80 ps, and the measurement time was 106 hours. The reconstructed density operator is shown in Fig. 2(c). An observed fidelity ${F}_{{\rm{swap}}}=\langle {\psi }^{-}|{\hat{\rho }}_{{\rm{swap}}}|{\psi }^{-}\rangle$ and the entanglement of formation (EoF)^24^ were 0.84 ± 0.04 and 0.82 ± 0.10, respectively, which indicates that a high-fidelity entanglement swapping is realized by using asynchronous polarization entangled photon sources and time-resolved coincidence measurement. We also estimated the maximized fidelity by the local phase shift as ${F^{\prime} }_{{\rm{swap}}}={{\rm{\max }}}_{-\pi \le \theta \le \pi }\langle {\psi }_{\theta }^{-}|{\hat{\rho }}_{{\rm{swap}}}|{\psi }_{\theta }^{-}\rangle =0.93\pm 0.04$ with *θ* = −0.62 rad, where $|{\psi }_{\theta }^{-}\rangle \,:=\,(|{\rm{H}}{\rm{V}}\rangle -{e}^{i\theta }|{\rm{V}}{\rm{H}}\rangle )/\sqrt{2}$.

### GHZ state generation

**Figure 3:**
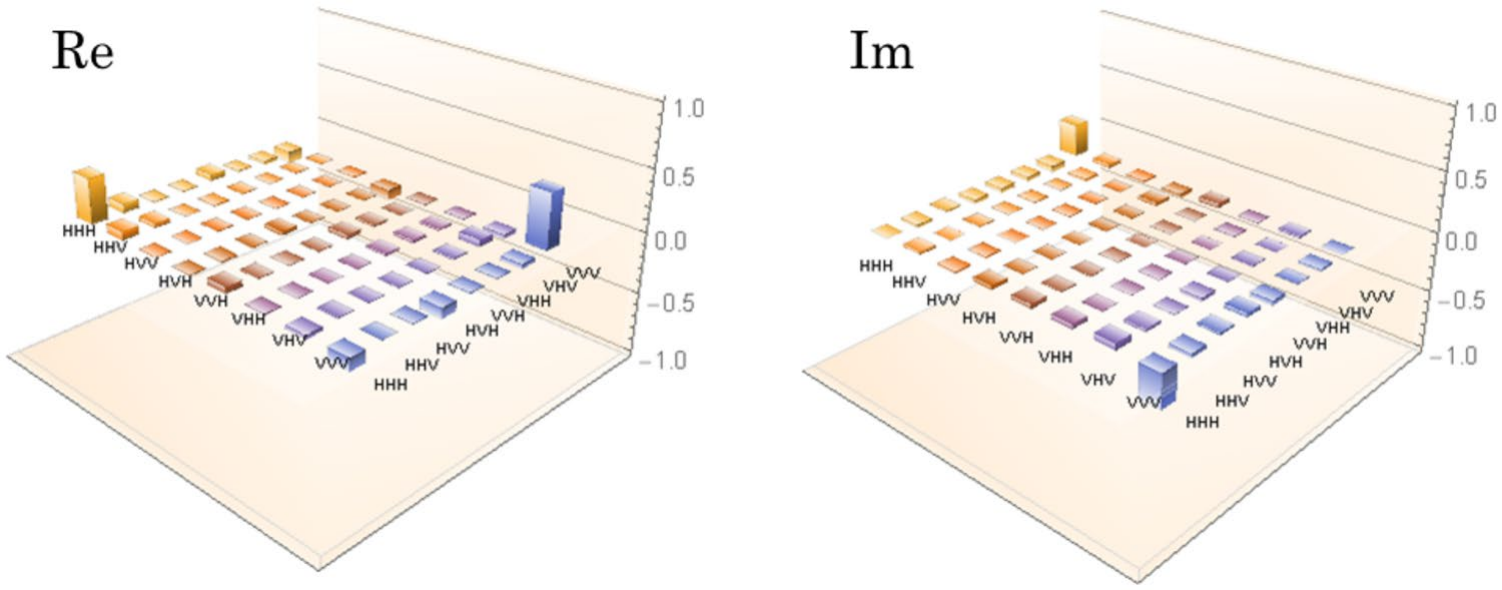
The real part and imaginary part of the density operators of ${\hat{\rho }}_{{\rm{GHZ}}}$.

With a similar setup to the one shown in Fig. 1, we demonstrated the generation of three-photon GHZ state using asynchronous photon sources at telecom wavelengths. GHZ state can be generated by the quantum parity check (QPC)^25^ on one half of the photon pair forming |*ϕ*^+^〉 and a D-polarized ancillary photon^26^. For this purpose, we changed the setup in Fig. 1 as follows: (1) We used the photon pair source B for generating a V-polarized photon pair by using only counterclockwise pump beam. A V-polarized photon in mode 3 is transformed to a D-polarized photon by using a half wave plate (HWP). (2) We replaced the HBS by a PBS in order to perform the QPC on photons in modes 3 and 4. When we detect a V-polarized photon at D2, a D-polarized photon is heralded in mode 3. When the photon in mode 3 is heralded by photon detection at D2 and the photon pair is generated in mode 1 and 4, the QPC is performed on photons in modes 3 and 4. By postselecting the events where all detectors click, the polarization state in modes 1, 3′ and 4′ ideally becomes the three-photon GHZ state $|{\rm{GHZ}}\rangle =(|{\rm{HHH}}\rangle +|{\rm{VVV}}\rangle )/\sqrt{2}$ with success probability of 1/2. We perform the QST on them to reconstruct its density operator ${\hat{\rho }}_{{\rm{GHZ}}}$. In this experiment, we set the pump power to be 5 mW on average for achieving higher four-fold coincidence rate. The detection rates of ${\hat{\rho }}_{A}$ and the photon pairs generated by the photon source B were 1.3 × 10^4^ Hz and 7.4 × 10^3^ Hz, respectively, with *τ*_w_ = 80 ps. The four-fold coincidence rate was 131 counts/hour and the measurement time was 87 hours. The reconstructed density operator (${\hat{\rho }}_{{\rm{GHZ}}}$) is shown in Fig. 3. The imaginary part arises from the non-zero relative phase between the H-polarized and V-polarized photons. We estimated the fidelity maximized by the local phase shift *F*_GHZ_ defined by ${F}_{{\rm{GHZ}}}={{\rm{\max }}}_{-\pi \le \theta \le \pi }\langle {{\rm{GHZ}}}_{\theta }|{\hat{\rho }}_{{\rm{GHZ}}}|{{\rm{GHZ}}}_{\theta }\rangle$, where $|{{\rm{GHZ}}}_{\theta }\rangle \,:=(|{\rm{HHH}}\rangle +{e}^{i\theta }|{\rm{VVV}}\rangle )/\sqrt{2}$. We obtained *F*_GHZ_ = 0.70 ± 0.05. In order to verify that ${\hat{\rho }}_{{\rm{GHZ}}}$ is a genuine three-photon entangled state, we used the witness operator^27,28^

1 $${\mathscr{W}}=\frac{\hat{I}}{2}-|{{\rm{GHZ}}}_{\theta }\rangle \langle {{\rm{GHZ}}}_{\theta }|,$$

where $\hat{I}$ is the identity operator. We obtain ${{\rm{\min }}}_{-\pi \le \theta \le \pi }\,{\rm{Tr}}({\mathscr{W}}\,{\hat{\rho }}_{{\rm{GHZ}}})=\mathrm{1/2}-{F}_{{\rm{GHZ}}}=-0.20\pm 0.05 < 0$, which shows that ${\hat{\rho }}_{{\rm{GHZ}}}$ possess genuine three-photon entanglement. This result ensures that the multi-photon entangled state can be generated by using fully autonomous sources via a time-resolved measurement. Such a telecom-band multipartite entanglement source has various applications such as nonlocality test and quantum communication.

## Discussion

**Figure 4:**
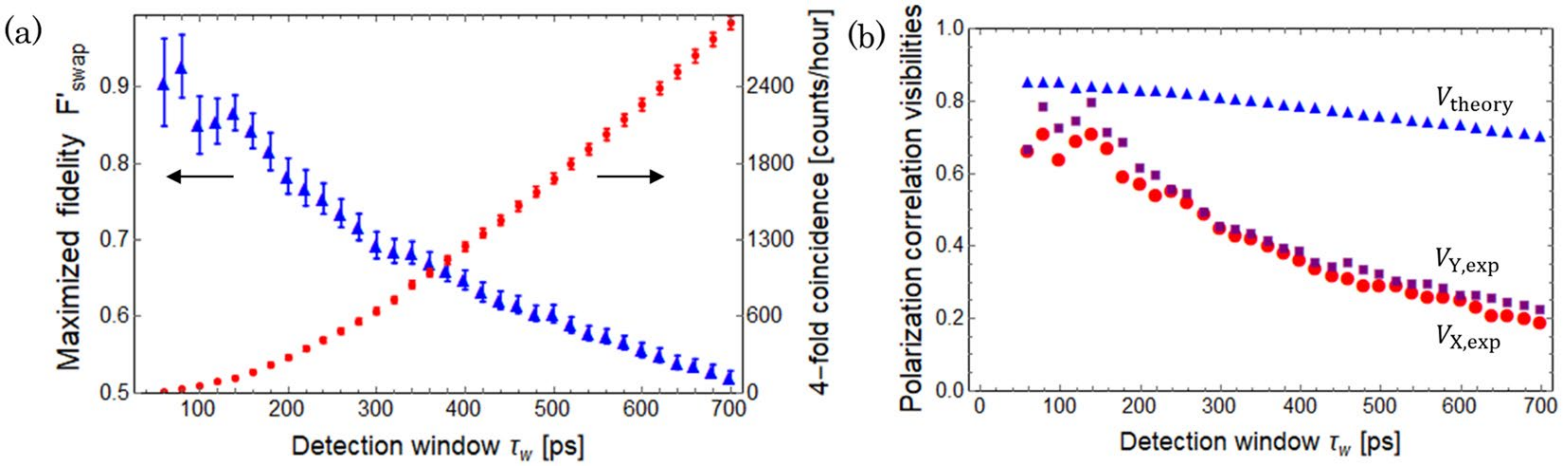
(**a**) ${F^{\prime} }_{{\rm{swap}}}$ (triangle) and the four-fold coincidence rate (circle) for various values of *τ*_w_. The four-fold coincidence rate is proportional to ${\tau }_{{\rm{w}}}^{2}$ for small *τ*_w_ because the two-fold coincidence probability of single photon pair is approximately proportional to *τ*_w_ in this region. On the other hand, for large *τ*_w_, the four-fold coincidence rate increases almost linearly because the two-fold coincidence between D1 and D3 (D4) does not increase much in this regime. (**b**) The detection time window *τ*_w_ dependencies of the theoretically-obtained polarization correlation visibility *V*_theory_ (triangle), and experimentally-obtained polarization correlation visibilities *V*_X,exp_ (circle) and *V*_Y,exp_ (square). *V*_X,exp_ and *V*_Y,exp_ decrease much faster than *V*_theory_ as we set the larger detection windows.

In the experiment using asynchronous sources, the fidelity of the final state and the four-fold coincidence rate depend on the width of each detection time window. Here we investigate the relation among them, and discuss the reason for the degradation of the fidelity of the swapped state. In asynchronous source experiment, employing small detection time windows is important for (1) satisfying the temporal indistinguishability of independent SPDC photons^10^ and (2) suppressing non-negligible continuous stray photons^14^. For investigating the influence of the width of each detection window *τ*_w_, we analysed the experimental data of entanglement swapping for various values of *τ*_w_ by post-processing of the record of timestamps. The trade off between the four-fold coincidence rate and ${F^{\prime} }_{{\rm{swap}}}$ can be seen in Fig. 4(a). We see that the ${F^{\prime} }_{{\rm{swap}}}$ still retains 0.75 ± 0.02 even if we set *τ*_w_ = 230 ps ~ *τ*_c_, where the four-fold coincidence rate is about 400 counts/hour, which is almost two orders higher than the previous reported value^10^ with a similar fidelity. In addition, the four-fold coincidence rate reaches about 3000 counts/hour for *τ*_w_ = 700 ps with ${F^{\prime} }_{{\rm{swap}}} > 0.5$, which still retains entanglement^29^.

Next, we discuss the reason for the degradation of the fidelity of the swapped state by introducing polarization correlation visibilities which we define ${V}_{{\rm{X}}}:=|{P}_{{\rm{DA}}}-{P}_{{\rm{DD}}}|/({P}_{{\rm{DA}}}+{P}_{{\rm{DD}}})$ and ${V}_{{\rm{Y}}}:=|{P}_{{\rm{RL}}}-{P}_{{\rm{RR}}}|/({P}_{{\rm{RL}}}+{P}_{{\rm{RR}}})$, where *P*_*mn*_ is the coincidence probability of the two photons when the polarization of the measured photons is (*m*, *n*) = (D, A), (D, D), (R, L) and (R, R). Here A, R and L represent anti-diagonal, right circular and left circular polarization, respectively. We assume that the polarization correlation visibilities of the initial photon pairs in spatial modes (1, 4) and (2, 3) in Fig. 1 take the same value which we denote by *V*_in_. In the following, we estimate the influence of the multiple pair creation at the SPDC sources on the polarization correlation visibilities of the swapped photon pairs, by constructing a theoretical model including presence of those photons but still assuming perfect indistinguishablity. We define the coincidence probability between the two photons in modes 3 and 4 heralded by the photons in modes 2 and 1 with *m* and *n* polarizations by ${P^{\prime} }_{mn}$. Assuming that the heralding probabilities at D1 and D2 do not depend on the polarizations *m* and *n*, the polarization correlation visibilities of the swapped photon pairs is represented by using ${P^{\prime} }_{mn}$ as ${V}_{{\rm{X}}({\rm{Y}})}=|{P^{\prime} }_{{\rm{DA}}({\rm{RL}})}-{P^{\prime} }_{{\rm{DD}}({\rm{RR}})}|/({P^{\prime} }_{{\rm{DA}}({\rm{RL}})}+{P^{\prime} }_{{\rm{DD}}({\rm{RR}})})$. We assume that each frequency mode of the input photons in modes 3 and 4 is in a single mode, and they have perfect temporal mode matching. We also assume that, when the heralding signal is produced by an *m*-polarized photon in mode 2 or 1, the *m*-polarizaion mode (mainly including the heralded photon) and the opposite *m*′-polarizaion (including stray and accidental photons) in mode 3 or 4 are statistically independent and have no phase relations. We denote the intensity and the autocorrelation function of the heralded (*m*) polarization by *s* and ${g}_{s}^{\mathrm{(2)}}$, and those of the opposite (*m*′) polarization by *n* and ${g}_{n}^{\mathrm{(2)}}$. These values are assumed to be independent of *m*. The ratio *χ* of the intensities are related to *V*_in_ as $\chi \,:=n/s=\mathrm{(1}-{V}_{{\rm{in}}}\mathrm{)/(1}+{V}_{{\rm{in}}})$. We can then derive a simple relation among *V*_*X*(*Y*)_, ${g}_{{\rm{s}}}^{\mathrm{(2)}}$, ${g}_{{\rm{n}}}^{\mathrm{(2)}}$ and *χ* as

2 $${V}_{X(Y)}={V}_{{\rm{theory}}}:=\frac{{\mathrm{(1}-\chi )}^{2}}{{g}_{s}^{\mathrm{(2)}}+{\chi }^{2}{g}_{n}^{\mathrm{(2)}}+{\mathrm{(1}+\chi )}^{2}}.$$

The detailed calculation is described in Supplementary information. In our experimental setup, if we run an additional experiment only by using photon pair source A(B), we may be able to obtain ${g}_{s}^{\mathrm{(2)}}$ and ${g}_{n}^{\mathrm{(2)}}$ separately. Instead, we calculated the relevant quantity appearing in Eq. (2) from the experimental data gathered for the entanglement swapping. An additional assumption we adopted here is that ${g}_{n}^{\mathrm{(2)}}=2$ is satisfied regardless of whether or not a heralding signal is produced in the opposite polarization mode. From the data, when the measured polarization in mode 1 was D, we obtained the three-fold coincidence count *C*_134_ among D1, D3 and D4, the single detection count *N* at D1, two-fold coincidence count *S*_13_ between D1 and D3 and *S*_14_ between D1 and D4 by ignoring the data of D2. From these observed values, we can determine the values of ${g}_{{\rm{ex}}}^{\mathrm{(2)}\,}:={C}_{134}N/({S}_{13}{S}_{14})=({g}_{s}^{\mathrm{(2)}}+2\chi \mathrm{(2}+5\chi \mathrm{))/(1}+3\chi {)}^{2}$ (details are given in Supplementary information). Combined with Eq. (2), we obtain

3 $${V}_{{\rm{theory}}}=\frac{{\mathrm{(1}-\chi )}^{2}}{1-2\chi -7{\chi }^{2}+{\mathrm{(1}+3\chi )}^{2}{g}_{{\rm{ex}}}^{\mathrm{(2)}}}.$$

From the observed two-fold coincidence counts of the initial photon pairs for various polarization measurement settings, we also estimated *P*_*mn*_, *V*_in_ and *χ*. Substituting *χ* and ${g}_{{\rm{ex}}}^{\mathrm{(2)}}$ into Eq. (3), we obtain *V*_theory_ for various values of *τ*_w_ as shown in Fig. 4(b). We also estimated the polarization correlation visibilities of the swapped state *V*_X,exp_ and *V*_Y,exp_ by using the four-fold coincidence counts of the swapped states for various polarization measurement settings and *τ*_w_. In Fig. 4(b), when ${\tau }_{{\rm{w}}}\ll {\tau }_{{\rm{c}}}$, we see that the experimentally-obtained values of *V*_X,exp_ and *V*_Y,exp_ are in good agreement with *V*_theory_ obtained by the above theoretical model. This means that the main cause of the degradation of the swapped state is multiple photon emission of SPDC process. The reason for the small gap between *V*_X(Y),exp_ and *V*_theory_ is the local phase shift of the swapped state. When we increase the detection windows, we see that the mismatch between *V*_X(Y),exp_ and *V*_theory_ increases. We guess the main reason is that the signal photons spread over multiple modes when ${\tau }_{{\rm{w}}}\gg {\tau }_{{\rm{c}}}$.

## Conclusion

In conclusion, we have demonstrated the high-fidelity entanglement swapping and the first demonstration of generating telecom-band three-photon entangled state by using two independent photon pairs generated by SPDC process with cw pump light. The observed fidelities are *F*_swap_ = 0.84 ± 0.04 and *F*_GHZ_ = 0.70 ± 0.05 with *τ*_w_ = 80 ps, which are as high as those observed in pulsed (synchronized) regime. In addition we investigated relation among *τ*_w_, the maximized fidelity and four-fold coincidence rate. We revealed that the four-fold coincidence rate becomes 400 counts/hour with the maximized fidelity of 0.75 ± 0.02 if we set *τ*_w_ ~ *τ*_c_ for entanglement swapping. For further enhancement of the four-fold coincidence rate without degrading the fidelity of the final state, the wavelength division multiplexing is effective^30,31^. For instance, when the width of emission spectrum of SPDC is several tens of nanometer, we can utilize several thousand frequency modes with the current filter band width, which will drastically improve detection rate of photon pairs as long as the linewidth of the pump laser is less than the width of the frequency bin. We believe that our results will be useful for many applications for a synchronization-free long-distance quantum communication.

## Electronic supplementary material

Supplementary information
