# Supplementary material for: High-fidelity entanglement swapping and generation of three-qubit GHZ state using asynchronous telecom photon pair sources
Source: Sci Rep. 2018 Jan 23;8:1446. doi: 10.1038/s41598-018-19738-8 (PMC5780511; doi:10.1038/s41598-018-19738-8)
Supplement: Supplementary file 1 — Supplementary information [file 41598_2018_19738_MOESM1_ESM.pdf]

Supplementary information of “High-fidelity entanglement swapping and generation of three-qubit GHZ state using asynchronous telecom photon pair sources”

Yoshiaki Tsujimoto[1,2*], Motoki Tanaka[1], Nobuo Iwasaki[1], Rikizo Ikuta[1], Shigehito Miki[3], Taro Yamashita[3], Hirotaka Terai[3], Takashi Yamamoto[1], Masato Koashi[4], and Nobuyuki Imoto[1]

[1]Graduate School of Engineering Science, Osaka University, Toyonaka, Osaka 560-8531, Japan,
[2]Advanced ICT Research Institute, National Institute of Information and Communications Technology (NICT), Koganei, Tokyo 184-8795, Japan,
[3]Advanced ICT Research Institute, National Institute of Information and Communications Technology (NICT), Kobe 651-2492, Japan,
[4]Photon Science Center, The University of Tokyo, Bunkyo-ku, 113-8656, Japan,
*tsujimoto@nict.go.jp

We derive a relation among the polarization correlation visibilities of the swapped photon pairs $V_{X(Y)} = |P'_{\mathrm{DA(RL)}} - P'_{\mathrm{DD(RR)}}|/(P'_{\mathrm{DA(RL)}} + P'_{\mathrm{DD(RR)}})$, the autocorrelation functions, and parameter $\chi := n/s = (1-V_{\mathrm{in}})/(1+V_{\mathrm{in}})$. We define creation operators of the input and the output light of the HBS as $\hat{a}^\dagger_{ik}$ and $\hat{b}^\dagger_{jk}$, respectively, where $i = 3,4$, $j = 3',4'$, $k = \mathrm{H,V,D,A,R,L}$. As in our experiment, we assume that the transmittance η_i of the system including the detection efficiency of Di for $i = 3,4$ is much less than 1 such that the detection probabilities are proportional to the photon number in the detected mode. The coincidence probability P' between D3 and D4 for a general input to the HBS is given by

$$P' = \eta_3\eta_4\langle \hat{b}^\dagger_{3'\mathrm{V}}\hat{b}^\dagger_{4'\mathrm{H}}\hat{b}_{3'\mathrm{V}}\hat{b}_{4'\mathrm{H}}\rangle \tag{S1}$$

For calculating P'_{DD} and P'_{DA}, we rewrite it in terms of input operators with polarizations D and A as $\hat{a}^\dagger_{i\mathrm{H}} = (\hat{a}^\dagger_{i\mathrm{D}} + \hat{a}^\dagger_{i\mathrm{A}})/\sqrt{2}$ and $\hat{a}^\dagger_{i\mathrm{V}} = (\hat{a}^\dagger_{i\mathrm{D}} - \hat{a}^\dagger_{i\mathrm{A}})/\sqrt{2}$. Using a unitary operator $\hat{U}$ of the HBS satisfying $\hat{U}\hat{b}^\dagger_{3'k}\hat{U}^\dagger = (\hat{a}^\dagger_{3k} + \hat{a}^\dagger_{4k})/\sqrt{2}$ and $\hat{U}\hat{b}^\dagger_{4'k}\hat{U}^\dagger = (\hat{a}^\dagger_{3k} - \hat{a}^\dagger_{4k})/\sqrt{2}$, Eq. (S1) is transformed into

$$\begin{aligned} P' &= \frac{1}{16}\eta_3\eta_4\langle\{(\hat{a}^\dagger_{3\mathrm{D}} - \hat{a}^\dagger_{4\mathrm{A}}) + (\hat{a}^\dagger_{4\mathrm{D}} - \hat{a}^\dagger_{3\mathrm{A}})\}\{(\hat{a}^\dagger_{3\mathrm{D}} - \hat{a}^\dagger_{4\mathrm{A}}) - (\hat{a}^\dagger_{4\mathrm{D}} - \hat{a}^\dagger_{3\mathrm{A}})\} \\ &\qquad \{(\hat{a}_{3\mathrm{D}} - \hat{a}_{4\mathrm{A}}) + (\hat{a}_{4\mathrm{D}} - \hat{a}_{3\mathrm{A}})\}\{(\hat{a}_{3\mathrm{D}} - \hat{a}_{4\mathrm{A}}) - (\hat{a}_{4\mathrm{D}} - \hat{a}_{3\mathrm{A}})\}\rangle & \text{(S2)} \\ &= \frac{1}{16}\eta_3\eta_4(\langle \hat{a}^\dagger_{3\mathrm{D}}\hat{a}^\dagger_{3\mathrm{D}}\hat{a}_{3\mathrm{D}}\hat{a}_{3\mathrm{D}}\rangle + \langle \hat{a}^\dagger_{3\mathrm{A}}\hat{a}^\dagger_{3\mathrm{A}}\hat{a}_{3\mathrm{A}}\hat{a}_{3\mathrm{A}}\rangle \\ &\qquad + \langle \hat{a}^\dagger_{4\mathrm{D}}\hat{a}^\dagger_{4\mathrm{D}}\hat{a}_{4\mathrm{D}}\hat{a}_{4\mathrm{D}}\rangle + \langle \hat{a}^\dagger_{4\mathrm{A}}\hat{a}^\dagger_{4\mathrm{A}}\hat{a}_{4\mathrm{A}}\hat{a}_{4\mathrm{A}}\rangle + 4(\langle \hat{a}^\dagger_{3\mathrm{D}}\hat{a}_{3\mathrm{D}}\rangle\langle \hat{a}^\dagger_{4\mathrm{A}}\hat{a}_{4\mathrm{A}}\rangle + \langle \hat{a}^\dagger_{3\mathrm{A}}\hat{a}_{3\mathrm{A}}\rangle\langle \hat{a}^\dagger_{4\mathrm{D}}\hat{a}_{4\mathrm{D}}\rangle)), & \text{(S3)} \end{aligned}$$

where we have used the assumption of independence between the D and A polarizations. For calculation of P'_{DD}, we substitute $\langle \hat{a}^\dagger_{3D}\hat{a}_{3D}\rangle = \langle \hat{a}^\dagger_{4D}\hat{a}_{4D}\rangle = s$, $\langle \hat{a}^\dagger_{3A}\hat{a}_{3A}\rangle = \langle \hat{a}^\dagger_{4A}\hat{a}_{4A}\rangle = n$, $\langle \hat{a}^\dagger_{3\mathrm{D}}\hat{a}^\dagger_{3\mathrm{D}}\hat{a}_{3\mathrm{D}}\hat{a}_{3\mathrm{D}}\rangle = \langle \hat{a}^\dagger_{4\mathrm{D}}\hat{a}^\dagger_{4\mathrm{D}}\hat{a}_{4\mathrm{D}}\hat{a}_{4\mathrm{D}}\rangle = s^2 g_s^{(2)}$, and $\langle \hat{a}^\dagger_{3\mathrm{A}}\hat{a}^\dagger_{3\mathrm{A}}\hat{a}_{3\mathrm{A}}\hat{a}_{3\mathrm{A}}\rangle = \langle \hat{a}^\dagger_{4\mathrm{A}}\hat{a}^\dagger_{4\mathrm{A}}\hat{a}_{4\mathrm{A}}\hat{a}_{4\mathrm{A}}\rangle = n^2 g_n^{(2)}$, leading to

$$P'_{\mathrm{DD}} = \frac{1}{8}\eta_3\eta_4(s^2 g_s^{(2)} + n^2 g_n^{(2)} + 4sn). \tag{S4}$$

For P'_{DA}, substituting $\langle \hat{a}^\dagger_{3D}\hat{a}_{3D}\rangle = \langle \hat{a}^\dagger_{4A}\hat{a}_{4A}\rangle = s$, $\langle \hat{a}^\dagger_{3A}\hat{a}_{3A}\rangle = \langle \hat{a}^\dagger_{4D}\hat{a}_{4D}\rangle = n$, $\langle \hat{a}^\dagger_{3\mathrm{D}}\hat{a}^\dagger_{3\mathrm{D}}\hat{a}_{3\mathrm{D}}\hat{a}_{3\mathrm{D}}\rangle = \langle \hat{a}^\dagger_{4\mathrm{A}}\hat{a}^\dagger_{4\mathrm{A}}\hat{a}_{4\mathrm{A}}\hat{a}_{4\mathrm{A}}\rangle = s^2 g_s^{(2)}$, and $\langle \hat{a}^\dagger_{3\mathrm{A}}\hat{a}^\dagger_{3\mathrm{A}}\hat{a}_{3\mathrm{A}}\hat{a}_{3\mathrm{A}}\rangle = \langle \hat{a}^\dagger_{4\mathrm{D}}\hat{a}^\dagger_{4\mathrm{D}}\hat{a}_{4\mathrm{D}}\hat{a}_{4\mathrm{D}}\rangle = n^2 g_n^{(2)}$, we have

$$P'_{\mathrm{DA}} = \frac{1}{8}\eta_3\eta_4(s^2 g_s^{(2)} + n^2 g_n^{(2)} + 2(s^2 + n^2)). \tag{S5}$$

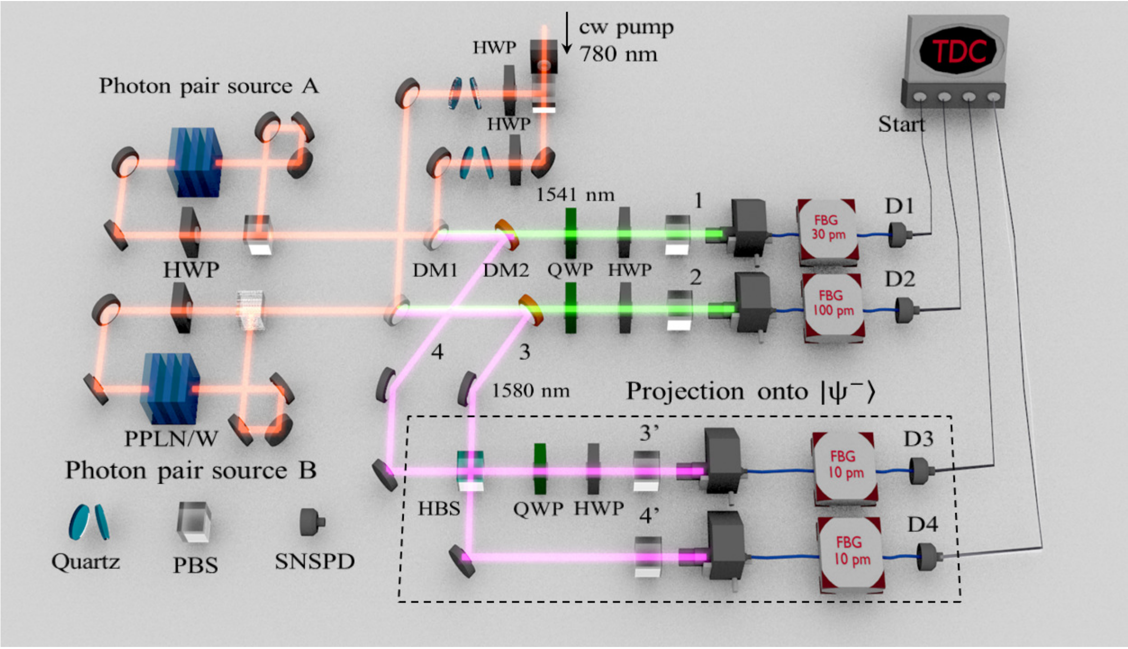

Figure S1. Experimental setup for entanglement swapping. The projective measurements on V polarization in mode 3' and H polarization in mode 4' are performed.

Thus the polarization correlation visibility V_X is expected to be

$$V_\mathrm{X} = V_\mathrm{theory} = \frac{(1-\chi)^2}{g_s^{(2)} + \chi^2 g_n^{(2)} + (1+\chi)^2}, \tag{S6}$$

where $\chi = n/s = (1-V_\mathrm{in})/(1+V_\mathrm{in})$. This formula happens to take the same form as the visibility of $V = 1 - P_0/P_\infty$ in a Hong-Ou-Mandel experiment in Ref. [1], which arises from the correspondence $P_0 = 4P'_\mathrm{DA}$ and $P_\infty = 2(P'_\mathrm{DA} + P'_\mathrm{DD})$. For calculating P'_RR and P'_RL, using the relations $\hat{a}^\dagger_{i\mathrm{H}} = (\hat{a}^\dagger_{i\mathrm{R}} + \hat{a}^\dagger_{i\mathrm{L}})/\sqrt{2}$ and $\hat{a}^\dagger_{i\mathrm{V}} = (\hat{a}^\dagger_{i\mathrm{R}} - \hat{a}^\dagger_{i\mathrm{L}})/(\sqrt{2}i)$, we can also show that $V_\mathrm{Y} = V_\mathrm{theory}$ in a similar manner.

Next, we derive the relation among $g_\mathrm{s}^{(2)}$, $g_\mathrm{n}^{(2)}$ and $g_\mathrm{ex}^{(2)} := C_{134}N/(S_{13}S_{14})$. The single detection probability $P_{3(4)}$ at D3(D4) for general input to the HBS is given by

$$P_{3(4)} = \eta_{3(4)} \langle \hat{b}^\dagger_{3'\mathrm{V}(4'\mathrm{H})} \hat{b}_{3'\mathrm{V}(4'\mathrm{H})} \rangle \tag{S7}$$

$$= \frac{1}{4}\eta_{3(4)} (\langle \hat{a}^\dagger_{3\mathrm{D}}\hat{a}_{3\mathrm{D}} \rangle + \langle \hat{a}^\dagger_{4\mathrm{D}}\hat{a}_{4\mathrm{D}} \rangle + \langle \hat{a}^\dagger_{3\mathrm{A}}\hat{a}_{3\mathrm{A}} \rangle + \langle \hat{a}^\dagger_{4\mathrm{A}}\hat{a}_{4\mathrm{A}} \rangle). \tag{S8}$$

The two-fold coincidence probability P_{34} is given by Eq. (S3). When the measured polarization in mode 1 is D, we substitute $\langle \hat{a}^\dagger_{4D}\hat{a}_{4D} \rangle = s, \langle \hat{a}^\dagger_{3D}\hat{a}_{3D} \rangle = \langle \hat{a}^\dagger_{3A}\hat{a}_{3A} \rangle = \langle \hat{a}^\dagger_{4A}\hat{a}_{4A} \rangle = n$, $\langle \hat{a}^\dagger_{4\mathrm{D}}\hat{a}^\dagger_{4\mathrm{D}}\hat{a}_{4\mathrm{D}}\hat{a}_{4\mathrm{D}} \rangle = s^2 g_s^{(2)}$ and $\langle \hat{a}^\dagger_{3\mathrm{D}}\hat{a}^\dagger_{3\mathrm{D}}\hat{a}_{3\mathrm{D}}\hat{a}_{3\mathrm{D}} \rangle = \langle \hat{a}^\dagger_{3\mathrm{A}}\hat{a}^\dagger_{3\mathrm{A}}\hat{a}_{3\mathrm{A}}\hat{a}_{3\mathrm{A}} \rangle = \langle \hat{a}^\dagger_{4\mathrm{A}}\hat{a}^\dagger_{4\mathrm{A}}\hat{a}_{4\mathrm{A}}\hat{a}_{4\mathrm{A}} \rangle = n^2 g_n^{(2)}$ into Eq. (S8) and Eq. (S3), leading to

$$P_{3(4)} = \frac{1}{4}\eta_{3(4)}(s+3n) \tag{S9}$$

and

$$P_{34} = \frac{1}{16}\eta_3\eta_4 \left(s^2 g_s^{(2)} + 3n^2 g_n^{(2)} + 4n(s+n) \right). \tag{S10}$$

Hence $g_\mathrm{ex}^{(2)}$ is given by

$$g_\mathrm{ex}^{(2)} = \frac{P_{34}}{P_3 P_4} = \frac{C_{134}N}{S_{13}S_{14}} = \frac{g_s^{(2)} + 3\chi^2 g_n^{(2)} + 4\chi(1+\chi)}{(1+3\chi)^2}. \tag{S11}$$

Since $\chi \ll 1$ is satisfied in our experiment, the right-hand side does not depend much on the value of $g_n^{(2)}$. We thus assume $g_n^{(2)} = 2$ here, which is consistent with the assumptions of the single mode and the polarization invariance adopted in our model. Then Eq. (S11) is represented by

$$g_{\text{ex}}^{(2)} = \frac{g_s^{(2)} + 2\chi(2+5\chi)}{(1+3\chi)^2}. \tag{S12}$$

Combining Eq. (S12) with Eq. (S6), we obtain

$$V_{\text{theory}} = \frac{(1-\chi)^2}{1-2\chi-7\chi^2+(1+3\chi)^2 g_{\text{ex}}^{(2)}}. \tag{S13}$$

References

1. Tsujimoto, Y. *et al.* High visibility Hong-Ou-Mandel interference via a time-resolved coincidence measurement. *Opt. Express* **25**, 12069–12080 (2017). .
